# Hand-held internet tablets for school-based data collection

**DOI:** 10.1186/1756-0500-1-52

**Published:** 2008-07-26

**Authors:** Simon J Denny, Taciano L Milfont, Jennifer Utter, Elizabeth M Robinson, Shanthi N Ameratunga, Sally N Merry, Theresa M Fleming, Peter D Watson

**Affiliations:** 1 Department of Community Paediatrics, Faculty of Medicine and Health Sciences, University of Auckland, Auckland, New Zealand; 2 Section of Epidemiology & Biostatistics, Faculty of Medicine and Health Sciences, University of Auckland, Auckland, New Zealand; 3 Department of Psychiatry, Faculty of Medicine and Health Sciences, University of Auckland, Auckland, New Zealand; 4 School of Psychology and Centre for Applied Cross-Cultural Research, Victoria University of Wellington, Wellington, New Zealand

## Abstract

**Background:**

In the last 20 years, researchers have been using computer self-administered questionnaires to gather data on a wide range of adolescent health related behaviours. More recently, researchers collecting data in schools have started to use smaller hand-held computers for their ease of use and portability. The aim of this study is to describe a new technology with wi-fi enabled hand-held internet tablets and to compare adolescent preferences of laptop computers or hand-held internet tablets in administering a youth health and well-being questionnaire in a school setting.

**Methods:**

A total of 177 students took part in a pilot study of a national youth health and wellbeing survey. Students were randomly assigned to internet tablets or laptops at the start of the survey and were changed to the alternate mode of administration about half-way through the questionnaire. Students at the end of the questionnaire were asked which of the two modes of administration (1) they preferred, (2) was easier to use, (3) was more private and confidential, and (4) was easier to answer truthfully.

**Results:**

Many students expressed no preference between laptop computers or internet tablets. However, among the students who expressed a preference between laptop computers or internet tablets, the majority of students found the internet tablets more private and confidential (p < 0.001) and easier to answer questions truthfully (p < 0.001) compared to laptop computers.

**Conclusion:**

This study demonstrates that using wi-fi enabled hand-held internet tablets is a feasible methodology for school-based surveys especially when asking about sensitive information.

## Findings

In the last 20 years, researchers have been using computer self-administered questionnaires to gather data on a wide range of adolescent health related behaviours[1,2]. A major advantage of computer-administered questionnaires is their potential to improve the quality of self-reported data collection. In comparison with pen and paper questionnaires, computer questionnaires generally increase the reporting of sensitive behaviours in young people[3]. The effect of computer administration on reporting of sensitive behaviours appears to be context specific and related to the perceived privacy of the mode of administration[2]. Computer administered questionnaires have been enhanced by the addition of an audio 'voice-over' and multimedia graphics[4]. With an audio 'voice-over', participants hear questions through headphones while at the same time they appear on screen. The addition of audio has been shown to further increase reporting of sensitive behaviours[2,5] and addresses issues of poor literacy, English as a second language, and visual impairment.

More recently, researchers have started using hand-held computers such as Personal Digital Assistants (PDAs) to collect survey information[6]. Hand-held computers have the advantage of being smaller and having greater portability. This may also result in more accurate data as the smaller size may mean that students perceive their answers to be more private and may be less inclined to be influenced by others around them [7]. Current limitations of hand-held computers in survey research include the small screen size and the lack of robust data handling software[6].

The aim of this study is to compare adolescent preferences of laptop computers or hand-held internet tablets in administering a youth health and well-being questionnaire, and to report on a new technology with wi-fi enabled hand-held internet tablets.

## Methods

Three schools from both rural and urban environments were asked to participate in the pilot study of Youth'07, a national youth health and well-being survey during August and September 2006. Two hundred and twenty-two students aged 12 to 17 years were randomly selected from the school roll based on the size of the school and invited to participate. Students and their parents were informed as to the purposes of the survey, given opportunities to ask questions and understood that their participation was voluntary. Participating students gave their consent to participate on the laptops or hand-held internet tablet at the beginning of the survey. Ethical approval was received from the University of Auckland Human subject ethics committee.

This survey of general health and well-being, included questions about sensitive risk behaviours and emotional well-being. The questionnaire covered areas such as home and family, school, emotional health concerns, sexual health, and substance use. A branched questionnaire design was used to limit exposure to sensitive questions for participants with no direct experience in these behaviours.

**Figure 1:**
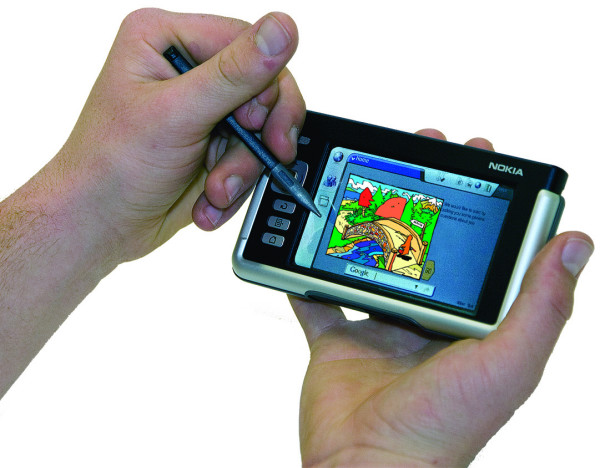
Hand-Held Internet Tablet (Nokia 770).

Both laptop computers (HP NX6120) and internet tablets (Nokia 770 Internet Tablet [8]) were used to administer the survey. The laptops and internet tablets were spread out to maximise student privacy. The internet tablet measures 135(5.3) × 78(3.1) × 14(0.5) mm(inches) with a high-resolution (800 × 480) touch screen (Figure 1). The questionnaire was web-based and transmitted to the computers using wi-fi technology. A closed wireless local area network was established using a laptop computer with Windows 2003 server bought into the classroom to administer the survey. The server laptop stored information from the student surveys on a database so that no information was lost if the laptop computers or internet tablets malfunctioned during the survey.

Students were assigned in groups of about 30 students to one of the sessions on the survey days. The survey questions and responses were displayed on the screen of the computers and were read out over headphones. Responses were read out only when the corresponding text on the screen was selected. Answers were recorded by students using a mouse for the laptops and a touch sensitive screen for the internet tablets. The survey was available in both English and Mâori languages with students able to toggle between these two languages. (Please see our website for a demonstration of the questionnaire interface and ability to toggle between languages [9].)

To compare internet tablets to laptops, participating students were randomly assigned to internet tablets or laptops at the start of the survey and were changed to the alternate mode of administration about half-way through the questionnaire. Students at the end of the questionnaire were asked which of the two modes of administration (1) they preferred, (2) was easier to use, (3) was more private and confidential, and (4) was easier to answer truthfully. Response options included 'laptop', 'nokia' or 'no difference'. Chi-square goodness-of-fit tests are used to test for equal proportions of students preferring laptop and internet tables amongst those who expressed a preference.

## Results

A total of 177 students (response rate 78%) took part in the survey. The mean age was 15.1 years (range 12 to 18 years), with similar participation by gender (52% female; 48% male). The preferences of students between laptop computers and internet tablets are shown in Table 1. Many students expressed no preference between laptop computers or internet tablets, especially when asked which modality they found it easier to answer questions truthfully (49%). There were no differences in students' preferred modality or ease of use between computer and internet tablets. However, among the students who expressed a preference between laptop computers or internet tablets, the majority of students found the internet tablets more private and confidential(p < 0.001) and easier to answer questions truthfully(p < 0.001) compared to laptop computers.

**Table 1:** Young people's preference to mode of administration of a youth health questionnaire. Laptop vs. Internet Tablet

Responses n (%)	No preference	Laptop	Internet Tablet	p value*
Which method do you prefer?	55 (31%)	61 (35%)	60 (34%)	0.93
Which method was easier to use?	65 (36%)	63 (36%)	49 (28%)	0.19
Which method was more private and confidential?	43 (24%)	26 (15%)	108 (61%)	<0.001
Which method was easier to answer truthfully?	86 (49%)	37 (21%)	54 (30%)	<0.001

* comparing proportions of students expressing a preference between laptop and internet tables.

Overall the average time taken to complete the questionnaire was 69 minutes with a mean of 66 minutes. The range was from 9 minutes to 117 minutes. The average number of questions answered was 451 (40%) out of a total of 1131 due to branching nature of the questionnaire. This number represents all questions with question sets and cumulative questions counted individually. For example a cumulative question with 20 response options is counted as 20 individual questions.

## Discussion

In this study of students responding to a health and well-being survey, among students who expressed a preference between laptop computers or internet tablets, internet tablets were reported to be more private and confidential and easier to answer truthfully compared with laptop computers. The small size of the hand-held computers and the relative close proximity of the laptop computers in the classroom setting may have influenced student's perceptions of privacy and confidentiality and therefore their ability to answer truthfully[10]. Students also found the hand-held computers as easy to use as laptops. These findings are consistent with recent research in school settings that have found high rates of acceptability of hand-held computers[6] with few differences in the rates of reporting of sensitive behaviours compared to laptop computers[11]. These findings add to current knowledge regarding the comparative attributes of interview modalities in youth surveys which have largely focussed on differences between pen and paper compared to computer administered questionnaires[7,12].

Previous research on the use of hand-held computers in school settings have utilised personal digital assistants or palm pilots. The advantage of internet tablets over personal digital assistants is a larger screen size allowing for more text and graphics to be displayed, including multiple stem questions. Using wi-fi enabled devices also means that the questionnaire could be administered and stored on a laptop server. This allows for the inclusion of audio files without limitations on memory on the hand-held devices and increases security of data handling.

## Conclusion

This study demonstrates that using wi-fi enabled hand-held internet tablets is a feasible methodology for school based surveys especially when asking about sensitive information. Further research needs to compare student's preferences and usability of internet-tablets with PDAs.

## Competing interests

The authors declare that they have no competing interests.

## Authors' contributions

SD, TM, TF, JU, SA, SM and PW participated in the development, implementation and management of this project and were involved in drafting the manuscript. ER participated in the statistical aspects of the design and analysis and helped with drafting of the manuscript.
